# Smartphone-Based Virtual Agents to Help Individuals With Sleep Concerns During COVID-19 Confinement: Feasibility Study

**DOI:** 10.2196/24268

**Published:** 2020-12-18

**Authors:** Pierre Philip, Lucile Dupuy, Charles M Morin, Etienne de Sevin, Stéphanie Bioulac, Jacques Taillard, Fuschia Serre, Marc Auriacombe, Jean-Arthur Micoulaud-Franchi

**Affiliations:** 1 USR 3413 SANPSY University of Bordeaux Bordeaux France; 2 Service de Médecine du Sommeil University Hospital of Bordeaux Bordeaux France; 3 SANPSY, USR 3413 Centre Nationale de la Recherche Scientifique Bordeaux France; 4 Ecole de psychologie Université Laval Quebec, QC Canada; 5 Centre d'étude des troubles du sommeil, Centre de recherche CERVO Institut universitaire en santé mentale de Québec Québec, QC Canada

**Keywords:** COVID-19, virtual agent, sleep disorders, technology acceptance, agent, sleep, smartphone, mobile phone, eHealth, feasibility, stress, app, intervention, behavior

## Abstract

**Background:**

The COVID-19 crisis and consequent confinement restrictions have caused significant psychosocial stress and reports of sleep complaints, which require early management, have increased during recent months. To help individuals concerned about their sleep, we developed a smartphone-based app called KANOPEE that allows users to interact with a virtual agent dedicated to autonomous screening and delivering digital behavioral interventions.

**Objective:**

Our objective was to assess the feasibility of this app, in terms of inclusion rate, follow-up rate, perceived trust and acceptance of the virtual agent, and effects of the intervention program, in the context of COVID-19 confinement in France.

**Methods:**

The virtual agent is an artificial intelligence program using decision tree architecture and interacting through natural body motion and natural voice. A total of 2069 users aged 18 years and above downloaded the free app during the study period (April 22 to May 5, 2020). These users first completed a screening interview based on the Insomnia Severity Index (ISI) conducted by the virtual agent. If the users were positive for insomnia complaints (ISI score >14), they were eligible to join the 2-stage intervention program: (1) complete an electronic sleep diary for 1 week and (2) follow personalized sleep recommendations for 10 days. We collected and analyzed the following measures: sociodemographic information, ISI scores and sleep/wake schedules, and acceptance and trust of the agent.

**Results:**

Approximately 76% (1574/2069) of the app users completed the screening interview with the virtual agent. The virtual agent was well accepted by 27.4% (431/1574) of the users who answered the acceptance and trust questionnaires on its usability, satisfaction, benevolence, and credibility. Of the 773 screened users who reported sleep complaints (ISI score >14), 166 (21.5%) followed Step 1 of the intervention, and only 47 of those (28.3%) followed Step 2. Users who completed Step 1 found that their insomnia complaints (baseline mean ISI score 18.56, mean ISI score after Step 1 15.99; *P*<.001) and nocturnal sleep quality improved significantly after 1 week. Users who completed Step 2 also showed an improvement compared to the initial measures (baseline mean ISI score 18.87, mean ISI score after Step 2 14.68; *P*<.001). Users that were most severely affected (ISI score >21) did not respond to either intervention.

**Conclusions:**

These preliminary results suggest that the KANOPEE app is a promising solution to screen populations for sleep complaints and that it provides acceptable and practical behavioral advice for individuals reporting moderately severe insomnia.

## Introduction

The current COVID-19 crisis has led to massive public health interventions, resulting in the confinement of almost the entire human population worldwide [1]. However, recent studies suggest that confinement may induce several negative psychological effects, including post-traumatic stress symptoms, anxiety, depression, anger, and insomnia [2,3]. Notably, Voitsidis et al [4] showed that in a study comprising 2363 Greek subjects, almost 38% of them reported insomnia complaints during the confinement due to COVID-19, and these complaints were associated with a higher rate of depression. The use of tobacco and alcohol in association with depression and stress symptoms has also increased during the COVID-19 pandemic [5].

These findings confirm that the COVID-19 crisis has caused major psychosocial stress and that prolonged confinement is potentially an aggravating factor for sleep complaints and insomnia. Therefore, given the large number of individuals affected and the limited number of health care professionals available during the crisis, there is a need for innovative solutions to track and help individuals at risk of psychosocial stress.

Digital technologies play a significant role in the context of the ongoing pandemic and overwhelmed health care services. As suggested by many researchers [6,7] and governmental authorities [1], technologies such as social media, smartphone apps, telehealth, and big data have great potential to disseminate information as well as screen and remotely monitor the general population, including patients with COVID-19. Several apps have been deployed in recent times to assist with COVID-19 management (eg, STOP COVID in France [8]). However, only a few apps and technologies specifically address psychosocial stress induced by the COVID-19 crisis and confinement [9-11]. To our knowledge, no study has focused on insomnia complaints, despite the evidence that digital behavioral therapies are effective to treat insomnia [12-14].

Soon after confinement measures were effective in France on March 17, 2020, we launched the first social media campaign in affiliation with our hospital and university through major national radios and newspapers. This campaign focused on the risk of insomnia and the measures to evaluate and correct inappropriate sleep hygiene practices among people during the COVID-19 confinement. In addition to social media campaigns, we developed a free smartphone app to help individuals with sleep concerns in the context of the COVID-19 pandemic. 

Named KANOPEE, the program is based on our previous research on embodied conversational agents (ECAs), also called virtual agents, which may be defined as animated characters that can engage in face-to-face dialogue through verbal and nonverbal behaviors. We previously demonstrated that ECAs can deliver a clinical interview to diagnose not only sleep complaints but also addiction and depression in an autonomous, reliable, valid, and acceptable way [15-19], by fostering empathy and facilitating disclosure of negatively connoted topics. In addition, based on the existing tools and knowledge on digital therapies for insomnia [14], we developed a digital sleep diary to automatically quantify the user’s daily sleep patterns and sleep duration, and to establish personalized sleep interventions guided by the data collected through the app. 

We hypothesized that a virtual agent made available via a smartphone app would be efficient and acceptable not only in providing autonomous screening for insomnia complaints but also in establishing digital behavioral interventions to help the population during the COVID-19 crisis. Therefore, to test our hypothesis, we launched a proof-of-concept study during the COVID-19 confinement.

 

## Methods

### Description and Implementation of KANOPEE

KANOPEE was implemented using the same architecture as our previous ECAs [15-19], with C# programming language in Unity 3D software (Unity Technologies). The virtual agent, named Louise, interacts through natural voice and body motion, as recorded by a professional artist by using motion capture technologies (Optitrack suit for 3D animations of Louise’s body and GoPro for 3D animations of Louise’s face and her voice). The interaction scenario is predefined, using decision trees to adapt to the user’s answers.

**Figure 1:**
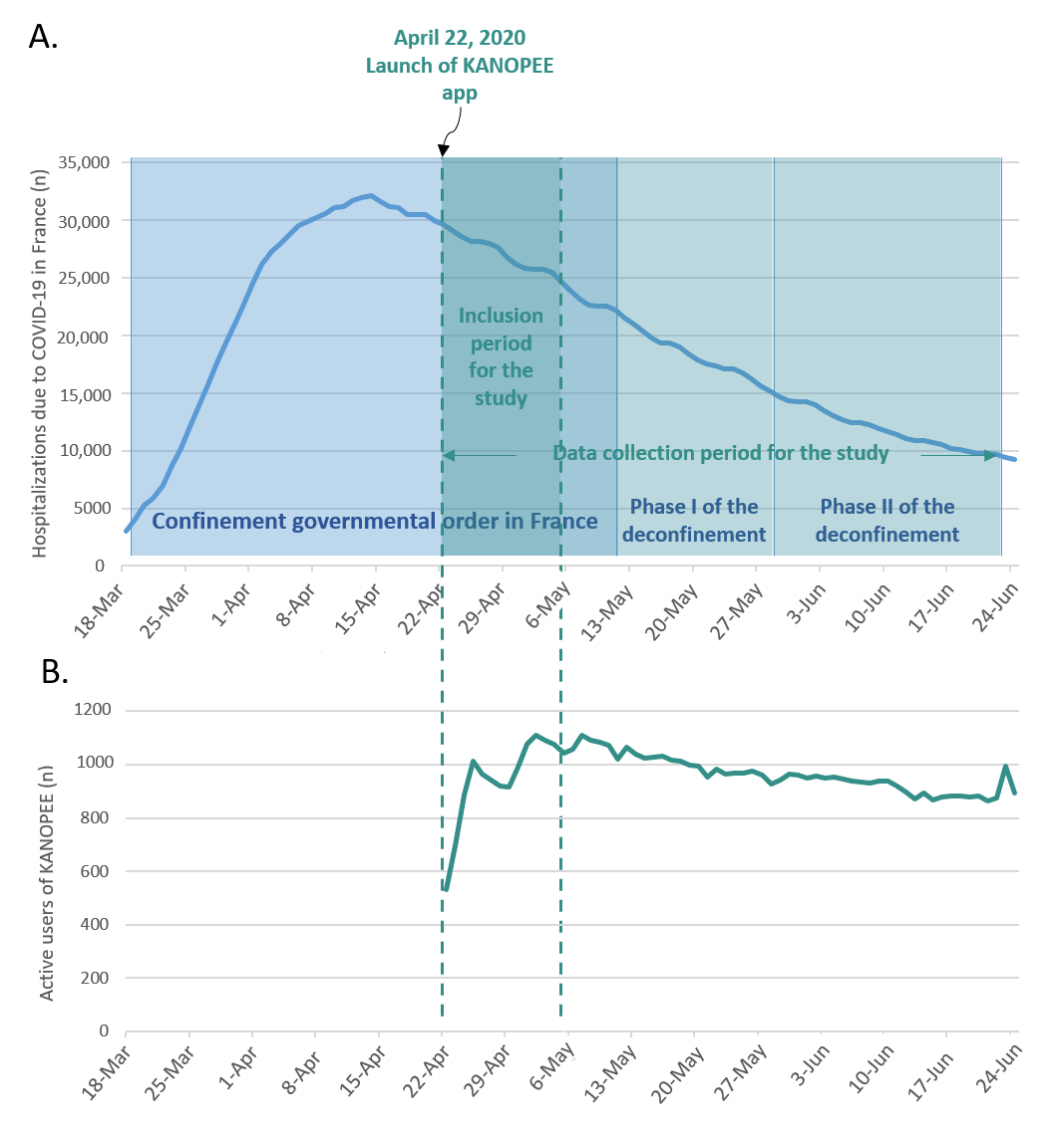
Conception and use of KANOPEE app. Chronological evolution of (A) the number of hospitalizations due to COVID-19 in France [20] and (B) the number of active users after the launch of the app across the confinement and deconfinement stages in France (based on data sourced from the Statistics page of the Google Play Store developer console for the app).

The app was made freely available on Google Play Store on April 22, 2020 (see Figure 1). After its launch, we organized a second media campaign presenting KANOPEE and showing how the app could help the French population to self-evaluate their sleep quality and provide practical solutions to manage insomnia. We analyzed the number of active app users across the confinement and deconfinement stages in France as well as hospitalizations due to COVID-19 during this time (Figure 1, [20])

### User Interaction

**Figure 2:**
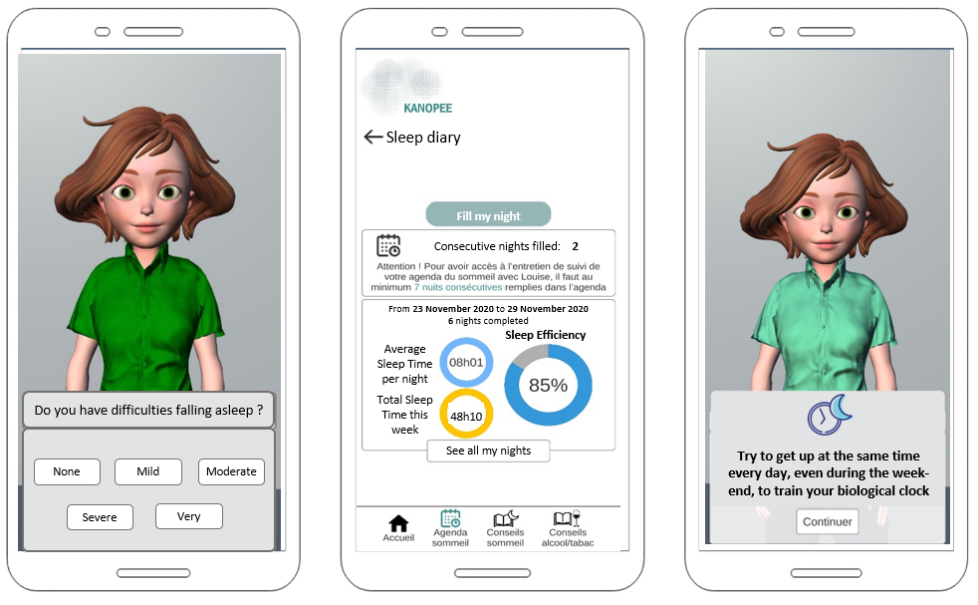
Illustrative references of KANOPEE app interfaces. From left to right, screenshot of (1) virtual agent Louise conducting an interview based on the Insomnia Severity Index, (2) sleep diary and visual feedbacks regarding user’s sleep patterns, and (3) a personalized sleep recommendation given by Louise during Interview 3.

The interaction scenario comprises of the following steps: first, during the screening interview (Interview 1), Louise introduces herself and administers the Insomnia Severity Index (ISI) [21] (see Figure 2, leftmost screen capture). Then, depending on the score, users are either provided with simple sleep hygiene recommendations (eg, follow usual wake up time, get exposed to sunlight in the morning, sleep in a quiet and dark room) if ISI score ≤14, or they are considered eligible to enter the intervention program if ISI score >14 [22]. Initially, users enter the “first step” of the intervention program, wherein they receive instructions to complete a sleep diary for 1 week in order to have a better understanding of their sleep patterns and to collect data about sleep indicators. Every morning, after filling in their sleep schedule, these users receive visual feedback on their sleep (eg, time spent in bed, total sleep time, and sleep efficiency; see Figure 2, middle screen capture). After completing the sleep diary for 7 days, they receive a follow-up interview request with Louise (Interview 2) wherein they learn about their sleep indicators from the previous week. At this point, they receive an ISI score for the second time. Next, the users can enter the “second step,” during which they are provided with personalized sleep recommendations for 10 days based on the sleep diary data and their answers on the ISI (see Figure 2, rightmost screen capture). Details of the conditions for providing personalized recommendations are listed in Textbox S1 (Multimedia Appendix 1). Thereafter, they can access another follow-up interview (Interview 3) and take the ISI a third time. Depending on the third ISI score, users can continue to use the app autonomously or, if their sleep complaints persisted (ie, ISI score >21), be referred to a sleep specialist in our hospital.

Throughout the process, all procedures and tools (ie, questionnaires and sleep diary) are introduced by the virtual agent in order to facilitate understanding among the users and increase their engagement. A demo video of the user interaction by Louise has been hosted on YouTube [23].

### Sociodemographics and Clinical Characteristics of Users

For the purpose of this study, users were selected for the analysis if they met the following inclusion criteria: (1) aged 18 years old and above and (2) had downloaded KANOPEE app before May 5, 2020, such that they had access to the 1-week intervention before the end of confinement period in France (ie, May 11, 2020). Their use of the app was recorded from April 22 until May 26.

After getting approval by the University and Hospital scientific committees, we obtained authorizations to be registered on the University Hospital register for General Data Protection Regulation (GDPR) approval by the French authorities—Commission nationale de l'informatique et des libertés (CNIL). Informed consent was obtained from all users downloading the app according to the GDPR and CNIL regulations.

**Figure 3:**
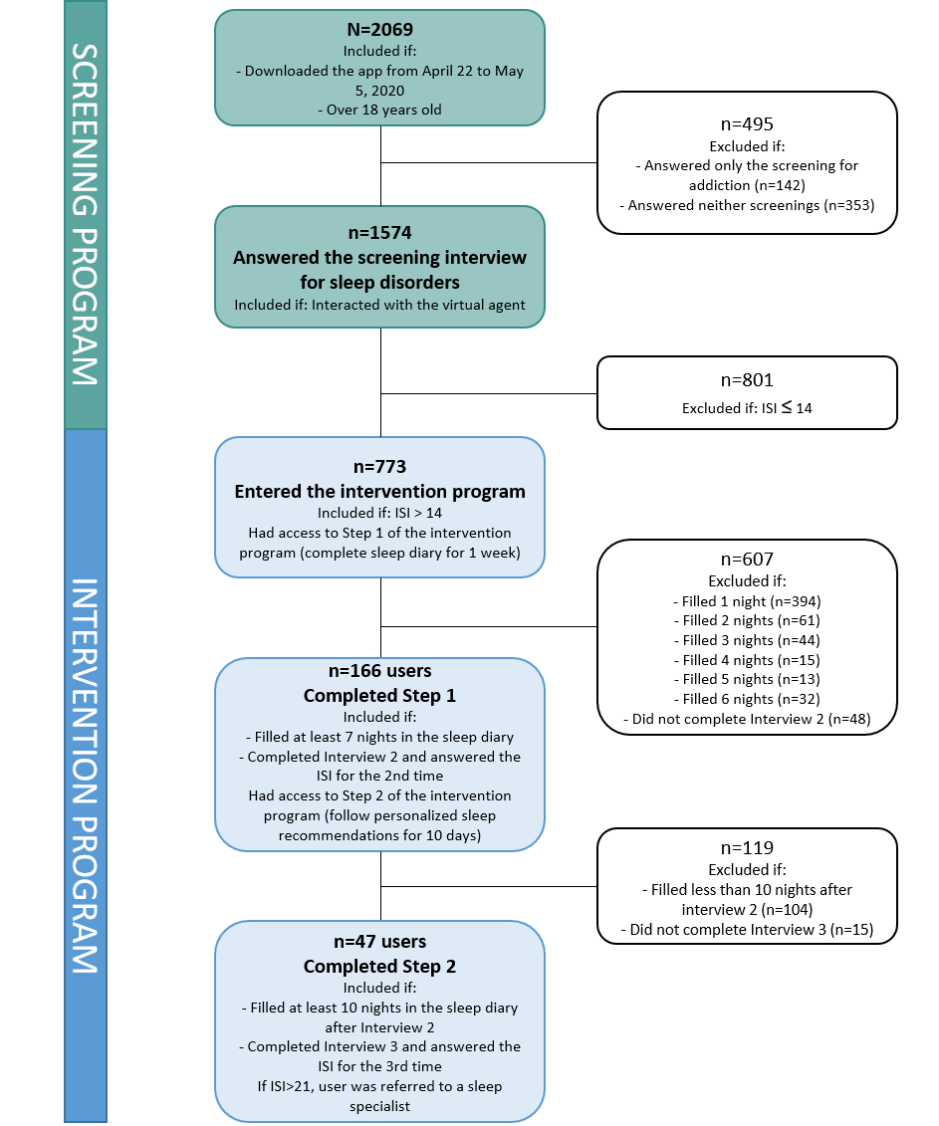
Flowchart of users included in the different steps of analyses.

Subgroups of users were then selected for more detailed analyses (see Figure 3). Specifically, users who answered the screening interview for sleep disorders were further examined to determine their eligibility for the next step of daily sleep monitoring, and users who reported insomnia complaints (ie, ISI score >14) entered the intervention program and were included in the analyses of outcomes and feasibility.

### Measures

#### Clinical Measures 

The ISI [21] is a 7-item self-report questionnaire that provides a global measure of perceived insomnia severity, with scores ranging from 0 to 28 (0-7: no clinical insomnia, 8-14: subthreshold insomnia, 15-21: insomnia of moderate severity, and 22-28: severe insomnia). The ISI has been previously validated and proven sensitive to changes in insomnia severity with treatment [22]. The ISI was used as a screening tool for assessing insomnia severity and as the primary outcome measure of treatment efficacy after the intervention.

Users who had an ISI score >14 were asked to complete a daily sleep diary in the app [24,25] throughout the intervention program. The following dependent variables were derived from users’ daily sleep diary entries: sleep onset latency (SOL, ie, how many minutes it takes to fall asleep, starting from the moment of intention to fall asleep), number of awakenings (NWAK), terminal wakefulness (TWAK, ie, the amount of awake time between the final wakefulness awakening and the time of getting out of bed), wake after initial sleep onset (WASO, ie, total amount of time awake during the night, excluding SOL and TWAK), total time spent in bed (TIB, ie, time starting from the moment of intention to fall asleep and concluding with the final arising), total sleep time (TST, ie, time actually spent sleeping) that was calculated from other self-reported variables (TIB−SOL− WASO−TWAK), and sleep efficiency (ie, percentage of time in bed spent asleep) that was calculated from other self-reported variables (TST/TIB × 100). 

Addictive behaviors (ie, alcohol and cigarette consumption) of users were evaluated through a clinical interview based on the CAGE (Cut down, Annoyed, Guilty, and Eye-opener) [26] and CDS-5 (Cigarette Dependence Scale, 5-item) questionnaires [27].

#### Acceptance and Trust Questionnaires

After the interviews with the virtual agent, users could complete 2 assessments on the app. The first assessment was the French version of the Acceptability E-scale (AES) [28,29] to measure acceptance of the KANOPEE app based on 2 subscores: usability (ie, the perceived ease of using the system or app) and satisfaction (ie, the perceived enjoyment of the use and usefulness of the system or app). The second assessment was the ECA trust questionnaire (ETQ) [18] that measures a user’s trust in a virtual agent based on 2 subdimensions: perceived credibility (ie*,* perception that the agent has the ability and the expertise to conduct a medical intervention) and benevolence (ie*,* perception that the agent is well-intentioned and will accurately take the user’s interests into account). Familiarity with technologies was also evaluated by a single question: “Are you familiar with computer technologies?” with the following 3 choices: “No,” “Moderately,” and “Yes,” which were scored as 0, 1, and 2, respectively.

### Statistical Analyses

Quantitative variables were expressed as means and SD, and qualitative variables were expressed as percentages. To compare 2 groups of users (eg, subclinical insomnia vs with moderate-to-severe insomnia), we performed 2-tailed Student *t* tests for continuous variables (eg*,* age, CDS score, and cigarettes smoked), and *χ*² tests for categorical variables (eg, gender, educational level, health care professional, and users adhering to the confinement). The data collected during the program were described using mean and SD values, and evolution of the measures over time was analyzed using repeated *t* tests. Acceptance (ie, usability and satisfaction subscores of the AES) and trust (ie, credibility and benevolence subscores of the ETQ) data were expressed using distributions and percentages. To investigate factors associated with acceptance and trust, we conducted univariate analyses with Pearson correlation analyses between 2 continuous variables (age, insomnia severity, and familiarity with technologies) and performed mean comparisons (*t* test or analysis of variance) to analyze the variation in AES and ETQ scores regarding categorical variables (gender and educational level). All analyses were performed using SPSS software (version 26; IBM Corp). 

## Results

### Characteristics of App Users

A total of 2069 users aged ≥18 years downloaded KANOPEE app and answered sociodemographic information (Table 1). Of these, 76% (1574/2069) of the users answered the screening interview for insomnia disorders. Most of these users were between 31 and 50 years old (mean 43.52, SD 13.94) and had a university degree. Furthermore, most users were in confinement due to the COVID-19 lockdown, and 5.6% (89/1574) were front-line health care professionals involved in the fight against COVID-19. Approximately half of the users (773/2069, 49.1%) who answered the screening interview for sleep disorders obtained an ISI score over the clinical threshold for insomnia (ie, ISI score >14). Compared to the users who answered the screening interview for sleep, those who chose not to were significantly older (*P*=.016) and predominantly male users (*P*=.001). Other factors remained nonsignificant between the 2 groups (see Table 1).

**Table 1:** Characteristics of KANOPEE users depending on their use of the app.

Characteristics		Values		Group comparison		*P* value
		Answered screening interview for sleep (n=1574)	Did not answer screening interview for sleep (n=495)	*t* test (*df*)	Chi-square (*df*)	
Age, mean (SD)		43.11 (13.8)	44.83 (14.4)	*2.40 (2067)* ^a^	N/A^b^	*.02*
**Age in years** **, n (%)**						
	18-30	338 (21.5)	99 (20)			
	31-50	763 (48.5)	220 (44.4)			
	51-65	366 (23.3)	131 (26.5)			
	>65	107 (6.8)	45 (9.1)			
**Gender, n (%)**				N/A	*10.71 (1)*	*.001*
	Female	1055 (67)	292 (59)			
	Male	519 (33)	203 (41)			
**Educational level, n (%)**				N/A	5.69 (*2*)	.13
	Middle school	318 (20.2)	83 (16.8)			
	High school	309 (19.6)	118 (23.8)			
	University degree	947 (60.2)	294 (59.4)			
Health care professionals, n (%)		89 (5.6)	28 (5.6)	N/A	0.00 (*1*)	.99
Confined due to COVID-19 lockdown, n (%)		1200 (76.2)	371 (74.9)	N/A	0.343 (*1*)	.50

^a^Italicized values indicate they are statistically significant.

^b^N/A: not applicable.

Users who answered the screening interview for sleep disorders (n=1574) were divided into 2 subgroups based on their performance on the ISI: scores ≤14 considered “with subclinical insomnia” and scores >14 considered “with moderate-to-severe insomnia” (Table 2).

Compared to users with subclinical insomnia, users with moderate-to-severe insomnia were younger (t_1576_=−3.03; *P*=.002), more educated (*χ^2^*_2_=12.14; *P*=.007), and more likely to be female (*χ^2^*_1_=31.91; *P*<.001). Interestingly, more users in confinement were found in the moderate-to-severe insomnia group (*χ^2^*_1_=8.86; *P*=.003), but we did not find evidence of a higher prevalence of insomnia among health care professionals. Users with moderate-to-severe insomnia smoked more cigarettes (t_734_=−4.03; *P*<.001) and obtained a higher score on the screening questionnaire for addiction to cigarettes (t_734_=3.41; *P*=.001) than those in the other groups.

**Table 2:** Characteristics of users depending on their sleep complaints.

Characteristics		Values		Group comparison		*P* value
		ISI^a^ score ≤14 (individuals with subclinical insomnia, n=801)	ISI score >14 (individuals with moderate to severe insomnia, n=773)	*t* test (*df*)	Chi-square (*df*)	
Age, mean (SD)		44.1 (14.3)	42.0 (13.1)	−*3.03 (1576)*^b^	N/A^c^	*.002*
**Age in years, n (%)**						
	18-30	166 (20.7)	172 (22.3)			
	31-50	372 (46.3)	391 (50.6)			
	51-65	194 (24.2)	172 (22.3)			
	>65	69 (8.7)	38 (4.9)			
**Gender, n (%)**				N/A	*31.91 (1)*	*.001*
	Female	484 (60.4)	571 (73.9)			
	Male	317 (39.6)	202 (26.1)			
**Educational level, n (%)**				N/A	*12.14 (2)*	*.007*
	Middle school	150 (18.6)	168 (21.7)			
	High school	139 (17.5)	170 (22.0)			
	University degree	512 (63.9)	435 (56.2)			
Health care professionals, n (%)		53 (6.6)	36 (4.7)	N/A	2.75 (*1*)	.097
Confined due to COVID-19 lockdown, n (%)		586 (73.1)	614 (79.4)	N/A	*8.86 (1)*	*.003*
Clinical insomnia, ISI score, mean (SD)		10.02 (3.42)	18.2 (2.74)	*52.12 (1572)*	N/A	*.001*
**Clinical insomnia subtype, n (%)**						
	No clinically significant insomnia (ISI score ≤7)	180 (22.5)	0 (0)			
	Subthreshold (ISI score 8-14)	621 (77.5)	0 (0)			
	Moderate (ISI score 15-21)	0 (0)	672 (86.9)			
	Severe (ISI score ≥21)	0 (0)	101 (13.1)			
CDS-5^d^, mean (SD)		4.53 (6.93)	6.45 (8.30)	*3.41 (734)*	N/A	*.001*
Daily number of cigarettes, mean (SD)		3.09 (6.40)	5.33 (8.68)	−*4.03 (734)*	N/A	*.001*
CAGE^e^, mean (SD)		0.65 (0.97)	0.78 (1.12)	1.66 *(734)*	N/A	.097
Daily number of drinks, mean (SD)		1.34 (2.09)	1.66 (3.18)	−1.62 *(734)*	N/A	.11

^a^ISI: Insomnia Severity Index.

^b^Italicized values indicate they are statistically significant.

^c^N/A: not applicable.

^d^CDS-5: Cigarette Dependence Scale-5 items.

^e^CAGE: Cut-down, Annoyed, Guilty, Eye-opener questionnaire.

### Trust and Acceptance of Virtual Agents

**Figure 4:**
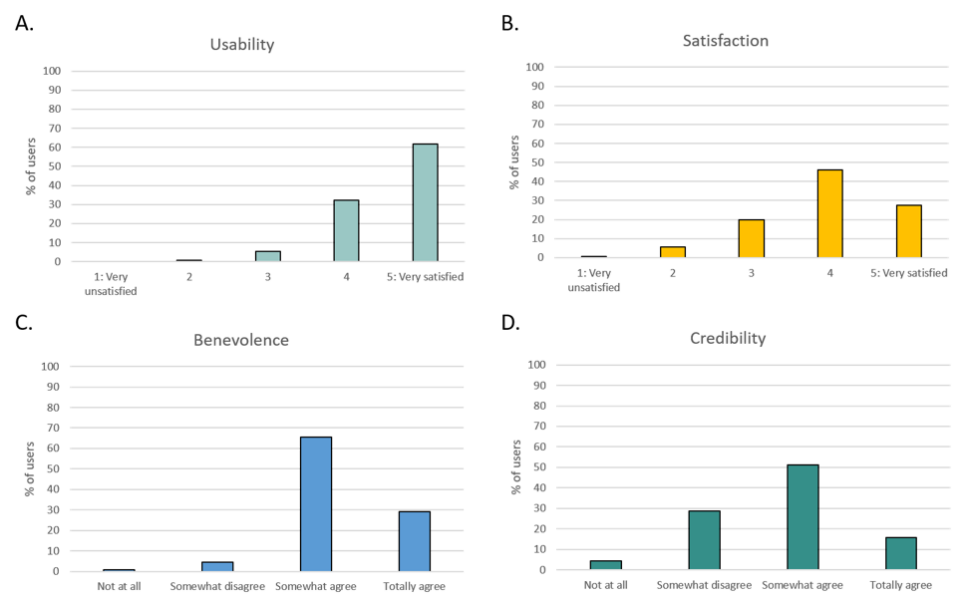
Distribution of usability, satisfaction, benevolence, and credibility perception of the virtual companion for sleep disorders (Louise). (A) Percentage of users’ rating for usability dimension (AES subscore), (B) percentage of users’ rating for satisfaction dimension (AES subscore), (C) percentage of users’ rating for benevolence dimension (ETQ subscore), and (D) percentage of users’ rating for credibility dimension (ETQ subscore).

In all, 431 of 1574 (27.4%) users answered the acceptance and trust questionnaires (Figure 4). Acceptance of the overall system (AES score) was rated very positively, with 61.7% (266/431) of users being “very satisfied” with the usability of the system, and 91.6% (395/431) of users rating the virtual agent more than 3 out of 5 for satisfaction. Regarding trust (ETQ score), Louise was perceived as trustworthy to perform medical interviews. Indeed, 94.6% (408/431) of users “somewhat agreed” or “totally agreed” that she was benevolent, and 67.05% (289/431) of users had a positive attitude towards her credibility (ie, rating of more than 1 out of 3). 

We found a negative correlation between age and credibility subscore on the ETQ (r=−.102; *P*=.034), suggesting that older individuals found Louise less credible than the younger ones. Age was not correlated with other dimensions of trust and acceptance. Similarly, gender and educational level of the users were not correlated with their attitude towards Louise. Regarding insomnia severity, there was a positive relationship between the severity and credibility of Louise (r=.125; *P*=.009), indicating that users with more severe insomnia complaints found her more credible. Lastly, we found significant correlations between users’ familiarity with technologies and their attitudes towards Louise: users more familiar with technologies found her more usable (r=.109; *P*=.024), more satisfactory (r=.128; *P*=.008), and more benevolent (r=.117; *P*=.015).

### Evolution of ISI Score and Nocturnal Sleep Indicators During the Intervention Program

**Figure 5:**
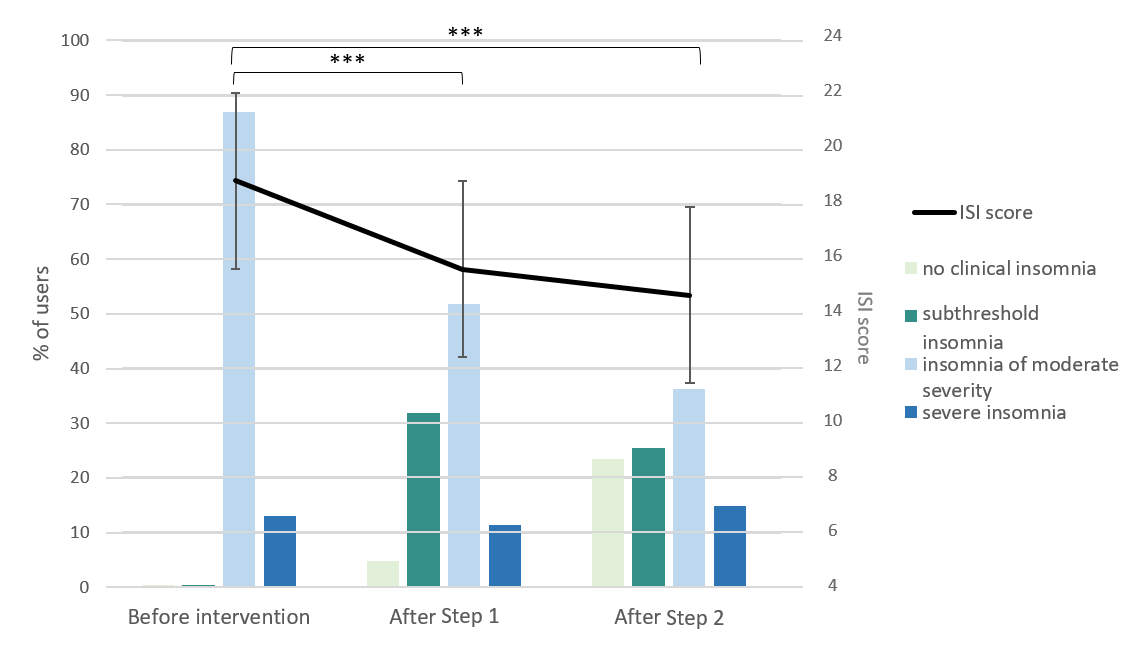
Distribution of users depending on the severity of their insomnia complaints (based on ISI scores) along the intervention program: Step 1: completed sleep diary; Step 2: followed personalized sleep recommendations. ISI: Insomnia Severity Index. Significance: *P<.05, **P<.001.

Among the 166 users who completed Step 1 of the intervention (ie, fill a sleep diary for 1 week and answer the ISI for the second time), the total ISI score (Figure 5) decreased compared to the baseline (baseline mean ISI score 18.56, mean ISI score after Step 1 15.99; t_165_=7.88; *P*<.001), with 36.7% (61/166) of users obtaining an ISI score below a clinically significant level (ie, ≤14) either corresponding to “no insomnia” (8/166, 4.8%) or to “subthreshold insomnia” (53/166, 31.9%). For the 47 users who completed Step 2 of the intervention, their ISI scores continued to decrease but did not reach a significant threshold (mean ISI score after Step 1 15.64, mean ISI score after Step 2 14.68; t_46_=1.42; *P*=.162). However, compared to the initial measure, a significant decrease was observed (baseline mean ISI score 18.87, mean ISI score after Step 2 14.68; t_46_=4.85; *P*<.001). Moreover, the proportion of users reporting low insomnia complaints increased, with a total of 48.9% (23/47) of users below a clinically significant level. Of note, 14.9% (7/47) of users still reported “severe insomnia” after Step 2, so they were referred to a sleep specialist.

Regarding nocturnal sleep patterns, we computed the mean scores of the first 2 nights filled in the sleep diary and the last 2 nights before receiving step 2 intervention in order to evaluate the evolution of sleep indicators during completion of Step 1 of the intervention program among the 166 users who completed the Step 1 (ie, filling in the sleep diary). Analyses of mean and SD values (see Table 3) suggest a reduction in TIB, SOL, and TWAK, and an increased sleep efficiency among this subgroup.

**Table 3:** Nocturnal sleep indicators of users completing Step 1 (n=166).

Sleep indicator	Value, mean (SD)		*t* test (*df*)	*P* value
	First 2 nights (1 & 2)	Last 2 nights (6 & 7)		
Time in bed, hh:mm:ss	08:56:45 (01:34:22)	08:40:27 (01:29:27)	*2.17 (165)^a^*	*.03*
Total sleep time, hh:mm:ss	06:04:32 (01:56:47)	06:21:30 (01:37:33)	−1.88 (*165*)	.06
Sleep efficiency, %	67.60 (20.25)	73.82 (17.33)	−*4.25* (*165*)	*.001*
Sleep onset latency, hh:mm:ss	01:31:38 (02:39:11)	00:58:37 (01:26.00)	*2.78* (*165*)	*.006*
Nocturnal awakenings, n	1.89 (1.57)	1.67 (1.32)	1.81 (*165*)	.07
Wake after initial sleep onset, hh:mm:ss	00:48:57 (00:55:48)	00:42:01 (00:52:58)	1.27 (*165*)	.21
Terminal wakefulness, hh:mm:ss	00:58:11 (01:09:42)	00:39:43 (0:39:57)	*3.31* (*165*)	*.001*

^a^Italicized values indicate they are statistically significant.

To measure the effect of completing Step 2 on sleep indicators, we computed the mean scores of the 7 nights before the users received personalized sleep recommendations and compared it to the mean scores of the 7 nights after they started Step 2. Mean and SD analyses among the 47 users who completed Step 2 suggest that WASO, NWAK, and TWAK decreased after Step 2, whereas TIB, TST, and sleep efficiency increased (see Table 4).

**Table 4:** Nocturnal sleep indicators of users completing Step 2 (n=47).

Sleep indicator	Value, mean (SD)		*t* test (*df*)	*P* value
	7 nights before Step 2	7 nights after Step 2		
Time in bed, hh:mm:ss	07:56:45 (01:18:51)	08:37:45 (00:50:21)	−5.35 (*46*)	.001
Total sleep time, hh:mm:ss	06:00:05 (01:24:29)	06:13:26 (01:30:16)	−2.02 (*46*)	.047
Sleep efficiency, %	68.47 (14.75)	72.36 (16.76)	−3.18 (*46*)	.002
Sleep onset latency, hh:mm:ss	00:59:22 (00:53:41)	01:08:50 (01:25:02)	−1.01 (*46*)	.32
Nocturnal awakenings, n	1.77 (1.24)	1.35 (0.97)	5.24 (*46*)	.001
Wake after initial sleep onset, hh:mm:ss	00:55:26 (00:52:03)	00:35:58 (00:32:57)	3.57 (*46*)	.001
Terminal wakefulness, hh:mm:ss	00:51:59 (00:42:15)	00:41:17 (00:32:22)	3.04 (*46*)	.003

## Discussion

### Principal Findings

Our results show, for the first time, the feasibility of using virtual agents in the context of a major health crisis to monitor insomnia symptoms and deliver assistance to the users through behavioral interventions. eHealth is a very rapidly growing field, and numerous solutions are particularly adapted to conditions such as confinement where human contacts must be limited. Several mobile apps use text-based chatbots for medical interviews, but the use of virtual agents (interacting through natural body motion and natural voice) is still sparse. We believe that these new empathic human-machine interfaces can reinforce acceptance of eHealth solutions.

More than 2000 people downloaded the KANOPEE app over the 11-day study period, with no technical errors reported by Google Play Store, indicating a higher inclusion rate than that reported in a previous study proposing digital cognitive behavioral therapy for insomnia [30]. This confirms the potential of digital technologies to provide access to clinical screening and behavioral intervention for insomnia for the general population [31]. Out of the 2069 users who downloaded KANOPEE, 1574 (76%) used it to self-evaluate their sleep. We noted that users who decided not to answer the screening interview for sleep were older and more likely to be male. This result might reflect more specific target populations such as young women—a group that is well known to report high levels of sleep complaints [32]. While the stress related to the COVID-19 confinement could explain why we obtained such a high app download rate, another potential explanation is the visual appeal of virtual agents to engage in digital interactions. Even individuals without significant sleep complaints (about 50% of the study sample) used our app and were interested in completing a sleep evaluation; this finding strongly indicates the need to develop apps for normal sleepers who want to improve their sleep hygiene and should be interpreted as a positive indicator for future sleep health campaigns. 

Acceptance of the virtual agent was a major challenge in this specific context, and we obtained very good results, similar to those reported previously with outpatients in a hospital [18]. Usability and benevolence were very well ranked by the app users, which confirms the empathic dimension of our virtual agents, even on smaller devices like smartphones. This is also a positive message to reinforce the use of virtual agents in eHealth technologies, which is a growing field of interest for medicine.

In the intervention program, 21.5% of the users reporting significant sleep complaints (ie, ISI score >14) completed a daily sleep diary for more than 7 days and consented to participate in Interview 2 (ie, they completed Step 1), and 28.3% of users followed behavioral interventions and completed the sleep diary for 10 more days. Interestingly, subjects completing Step 1 significantly improved their sleep over a brief period of time (ie, 7 days). We hypothesize that filling in the sleep diary and receiving daily feedback on their sleep efficiency score helped users to adjust their sleep schedule autonomously. Another possible explanation is that their insomnia symptoms decreased naturally over time, even though a reduction of time in bed for about half an hour suggests an active change. These findings are very encouraging for the use of electronic sleep diaries to promote sleep hygiene practices, a form of low-intensity sleep health intervention that could be beneficial at the more global population level. Users who completed their personalized intervention reported an improvement in nocturnal sleep, with a reduction of nocturnal awakenings and insomnia complaints, which suggests Step 2 was beneficial for a subgroup of individuals with more significant sleep complaints. Altogether, with completion rates of 76% for the initial evaluation and 28.3% for the personalized interventions in a selected population, we believe that our results open interesting perspectives for populational interventions and mirror the proposal of Berry et al [33] to set up trials in which large-scale interventions are offered simultaneously to different subgroups of patients. 

### Study Limitations

Nevertheless, this study has a few limitations. First, the very peculiar period of recruitment, during the COVID-19 confinement, makes our results preliminary. Future work needs to confirm, in a more “normal period of time,” the fact that KANOPEE can help individuals improve their insomnia complaints and sleep hygiene. Second, the drop-out rate was quite high. We were unable to determine why users did not follow the program until the end, and further study is therefore needed for a precise examination of usage (eg, frequency of use and errors made) and qualitative interviews with app users to unveil the reasons why they decided to drop out of KANOPEE.

Another limitation of our study is related to the fact that we did not note an improvement among the most severe users, which shows the limitations of nonhuman interventions. Because we did not explore all the possible comorbidities, we might have proposed to some users a solution that may be unsuitable to their health problems. Future studies could use detailed interviews that could help precisely select the ideal population to receive the interventions and refer the other users directly to sleep centers.

### Conclusions

Considering the above limitations, we believe that KANOPEE is a new promising tool in the field of eHhealth that could limit the number of individuals asking for consultations by general practitioners for moderate sleep complaints. Indeed, we believe this app can help in both ways: identifying individuals with insomnia complaints and providing brief and practical behavioral interventions. Further research is needed to test this app apart from the COVID-19 confinement period and on more specifically selected users.

## Appendix

Multimedia Appendix 1 Personalized sleep recommendations and conditions.

## Abbreviations

AES: Acceptability of e-health scale
CAGE: Cut-down, annoyed, guilty, eye-opener questionnaire
CDS-5: Cigarette Dependence Scale (5-item) questionnaire
CNIL: Commission nationale de l'informatique et des libertés
ECAs: embodied conversational agents
ETQ: ECA trust questionnaire
GDPR: General Data Protection Regulation
ISI: insomnia severity index
NWAK: number of awakenings
SOL: sleep onset latency
TIB: time spent in bed
TST: total sleep time
TWAK: terminal wakefulness
WASO: wake after initial sleep onset
