# Supplementary material for: Smartphone-Based Virtual Agents to Help Individuals With Sleep Concerns During COVID-19 Confinement: Feasibility Study
Source: J Med Internet Res. 2020 Dec 18;22(12):e24268. doi: 10.2196/24268 (PMC7752183; doi:10.2196/24268)
Supplement: Multimedia Appendix 1 [file jmir_v22i12e24268_app1.doc]

**Textbox 1:** Personalized sleep recommendations and conditions for personalization.

| **Personalized sleep recommendations** | |
| --- | --- |
| 1. Try to get up at the same time every day, even during the week-end, to train your biological clock.  2. Stay in bed for reasonable time in order to reinforce association bed-sleep: if you don’t sleep, get up!  2alt. If you are awake for more than 15min, get up; and get back to bed only when you feel sleepy.  3. In the morning expose yourself to sunlight or to other source of bright light (luminotherapy, screen) to improve functioning of your biological clock.  4. Physical activity will help to stabilize your biological clock. Ideally, do some sport 1h in the morning, and do not exercise 3 to 4 hours before going to bed.  5. Try to not change your sleep schedule, and do not stay in bed even if you experienced a bad night.  6. Moderate your consumption of stimulating beverages (coffee, sodas, energizer drinks): no more than 4 cups a day, and not after 2pm.  7. Go to bed only when you feel sleepy. Try not to read or eat in bed  8. In the evening, do not skip your meal, but avoid fat-rich dishes and favor starch foods, which help not feeling hungry at night.  9. Do not use your electronic devices (smartphone, TV, tablet) 1 to 2 hours before going to bed.  10. Accommodate your bedroom in conditions conducive to sleep: dark, quiet, and room temperature from 18°C to 20°C.  11. Be careful! You might be experiencing sleep deprivation, try to sleep at least 7h per night.  12. Before going to bed, try exercising abdominal breathing. | |
| **Conditions for personalization** | |
| If item 1 of ISI (falling asleep)=severe or very severe | Give recommendations 2, 3, 7, 12 |
| If item 2 of ISI (staying asleep)=severe or very severe, or if duration of nocturnal awakening > 15min | Give recommendations 2alt, 4, 5, 6, 10 |
| If SE > 85% and TST > 7h | Give recommendations 1, 3, 4, 5 |
| If SE > 85% and TST < 6h and ISI total score > 21 | Give recommendation 11 |
| Else | Give recommendations 1, 2, 3, 4, 6, 8, 9, 10 |
